# Investigation of Dissolution Mechanism and Release Kinetics of Poorly Water-Soluble Tadalafil from Amorphous Solid Dispersions Prepared by Various Methods

**DOI:** 10.3390/pharmaceutics11080383

**Published:** 2019-08-02

**Authors:** Tereza Školáková, Michaela Slámová, Andrea Školáková, Alena Kadeřábková, Jan Patera, Petr Zámostný

**Affiliations:** 1 Department of Organic Technology, University of Chemistry and Technology, Prague, Technická 5, 166 28 Prague 6, Czech Republic; 2 Department of Metals and Corrosion Engineering, University of Chemistry and Technology, Prague, Technická 5, 166 28 Prague 6, Czech Republic; 3 Department of Polymers, University of Chemistry and Technology, Prague, Technická 5, 166 28 Prague 6, Czech Republic

**Keywords:** solid dispersion, tadalafil, Wood’s apparatus, intrinsic dissolution rate, Weibull dissolution model, dissolution rate

## Abstract

The aims of this study were to investigate how the release of tadalafil is influenced by two grades of polyvinylpyrrolidone (Kollidon^®^ 12 PF and Kollidon^®^ VA 64) and various methods of preparing solid dispersions (solvent evaporation, spray drying and hot-melt extrusion). Tadalafil is poorly water-soluble and its high melting point makes it very sensitive to the solid dispersion preparation method. Therefore, the objectives were to make a comparative evaluation among different solid dispersions and to assess the effect of the physicochemical nature of solid dispersions on the drug release profile with respect to the erosion-diffusion mechanism. The solid dispersions were evaluated for dissolution profiles, XRD, SEM, FT-IR, DSC, and solubility or stability studies. It was found that tadalafil release was influenced by polymer molecular weight. Therefore, solid dispersions containing Kollidon^®^ 12 PF showed a faster dissolution rate compared to Kollidon^®^ VA 64. Tadalafil was released from solid dispersions containing Kollidon^®^ 12 PF because of the combination of erosion and diffusion mechanisms. The diffusion mechanisms were predominant in the initial phase of the experiment and the slow erosion was dissolution-controlling at the second stage of the dissolution. On the contrary, the tadalafil release rate from solid dispersions containing Kollidon^®^ VA 64 was controlled solely by the erosion mechanism.

## 1. Introduction

Poorly water-soluble drugs showing pharmacological activity are a common and ongoing issue for the pharmaceutical industry and associated with the complexity of the drug development [1,2,3]. It has been reported that approximately 40% of marketed drugs and 75% of active pharmaceutical ingredients under development are classified as practically insoluble in water [3].

Solid dispersions of such drugs in hydrophilic carriers have provided a promising possibility of improving their dissolution rate, and thereby absorption [4]. Basically, the solid dispersions are two-component systems which can improve drug wettability and bioavailability significantly by reducing the effective drug particle size to the absolute minimum, increasing the drug surface area, reducing its crystallinity, and increasing wettability by surrounding hydrophilic carriers due to their unique morphology [5,6,7,8]. They are very attractive for formulators due to well-known preparation processes and devices, high effectiveness, flexibility in designing the composition, or low batch-to-batch variability [6,9]. However, the ideal state of molecular dispersion may not be always achieved, and the solid dispersions may only approach the molecular level of an ideal solid solution. That is the case especially in formulations where the melting point of the drug exceeds the maximum processing temperature of the polymer, so that the dispersion is formed effectively by dissolving the drug in molten polymer or by introducing additional solvent, rather than by co-melting of the drug-polymer mixture. In such systems, the preparation method is likely to have strong impact on the apparent solubility and drug release. While different preparation techniques were reported in the literature for many systems, the link between the preparation method and the dissolution properties has not been clearly established, and therefore it requires further study [5].

The objective of this study was to investigate and to provide insights into how the drug release is influenced by various polymer carriers and methods of preparation. Tadalafil (TAD) solid dispersions formulated with two grades of polyvinylpyrrolidone (Kollidon^®^ 12 PF and Kollidon^®^ VA 64) were used as model system for this study. TAD was selected as a poorly soluble, hydrophobic drug, with a melting point around 300 °C, which is well above the processing temperature of common polymers used for the solid dispersion formulations. Therefore, it provides a very sensitive system for studying the effects of preparation methods on the dispersion properties and the subsequent dissolution behavior. At the same time, it represents a compound which was the subject of many formulation studies recently (see next section for the details) and thus this choice is relevant from the application point of view. The polymers were selected due to their widespread use in solid dispersion formulations. They are hydrophilic in nature, which have potential to change the crystalline drug to amorphous by solid dispersion technique, enhancing TAD wettability.

The specific goals to disclose within this paper include the comprehensive analysis of TAD solid dispersions, analysis of the dissolution profiles of different TAD formulations with respect to the erosion-diffusion mechanism, and a comparison of the dissolution behavior of TAD from physical mixtures and corresponding solid dispersions, as studied by different dissolution techniques like apparent intrinsic dissolution and flow-through cell apparatus. Those results are then linked to the preparation methods of solvent evaporation, spray drying, and hot-melt extrusion.

### 1.1. Theoretical Background

This section provides detailed background information summarizing the reported techniques used for improving the dissolution properties of TAD and dissolution phenomena in polymer matrix systems, which were separated from the general introduction for the sake of clarity, but which can help in understanding the methods and the discussed results, and provide necessary reference for state of the art.

#### 1.1.1. Techniques for Improving Dissolution Rate of TAD

Based upon aqueous solubility and dissolution parameters, the efficacy TAD used for the treatment of erectile dysfunction, pulmonary arterial hypertension, and now also for therapy in pyelonephritis can be limited by its highly hydrophobic particles [10,11]. The dissolution is considered as the first step in the absorption process and therefore, a critical disadvantage of TAD is its poor water solubility [12,13]. Since the pK*a* value of TAD is 16.68, it is a non-ionizable drug via the range of physiological pH. Therefore, neither ionization nor salt formation can be applied for the enhancement of its aqueous solubility and dissolution rate and thus, it was undertaken to develop effective methods for improvement of these properties [5,14]. The transformation of pure crystalline TAD into the amorphous form is considered to be troublesome [15]. Wlodarski et al. obtained the amorphous forms of TAD by amorphization methods and without excipients, i.e., by cryogenic grinding, ball milling, spray drying, freeze-drying and antisolvent precipitation. On the other hand, they also reveal that vitrification was an inappropriate method to convert to amorphous counterpart of crystalline TAD because of its decomposition at the melting point temperature. Their study also revealed that the techniques influence the apparent water TAD solubility insubstantially; however, disk intrinsic dissolution rate tests of amorphous TAD obtained by ball milling and spray drying did not improve in the rate of its dissolution [16]. Therefore, several technologies have been used to enhance the dissolution rate and solubility of TAD, including solid dispersion systems, e.g., in [3,6,17,18,19,20], the formulation of self-microemulsifying composition (SMEC) [11], self-nanoemulsifying drug delivery system (SNEDDS) [21], nanostructured lipid carriers (NLCs) [22], nanoparticles [13,23], cyclodextrin complexation [24], incorporation in microporous silica [25] or microemulsion system [17]. Of these formulations, however, solid dispersion is currently favored in the pharmaceutical industry [19].

Solid dispersion of TAD has been prepared using different methods, these include solvent evaporation method [8,10,12,18,19], spray drying [6,16,26], melting method [5,17], hot-melt extrusion [15], supercritical anti-solvent process [14,27], ball milling [26,27,28] or freeze-drying [3].

#### 1.1.2. Dissolution Phenomena in Polymer Matrix Systems

The polymeric matrix systems, such as hydrogel-based dosage forms, are commonly used for manufacturing sustained release drug delivery systems [29,30]. Solid dispersions can also be used for controlling drug release [31]. However, the drug release from such systems involves many phenomena which can contribute to the final progress of dissolution and therefore, the complete description is very complex and not completely understood [32,33,34]. The critical factors in the release of drugs from matrix system are clearly summarized and reported in [35]. Briefly, when the solid dosage forms, based on a hydrophilic polymer matrix, are immersed in a dissolution medium, the surface of the matrix is wetted, the water penetrates into the systems and polymer surface swells to form a gel leading to the polymer chain relaxation, drug dissolution, drug diffusion through the hydrated polymeric network, chain disentanglement of polymer, matrix erosion and moving boundaries [36,37]. Since the polymer undergoes a relaxation leading to its erosion, the diffusion is generally not the main mechanisms that control the drug release [38]. Moreover, three fronts, and of course corresponding boundaries, can be identified in the polymer-based drug delivery, i.e., polymer glassy-rubbery transition boundary (swelling front), solid drug-drug solution boundary (diffusion front) and swollen matrix-solvent boundary (erosion front) [39,40]. Therefore, the dissolution rate of drug is affected by the movement of these fronts and the mechanisms of release can operate simultaneously [38,39]. In other words, the rate of water uptake is widely associated with the position of the swelling front [41]. However, it can be expected that erosion front movement determines the kinetics whilst the diffusion front movement determines the rate of drug release [39]. Of course, some of the phenomena occurring during water uptake can also be observed in the matrix size (its size enlargement or reduction), e.g., the swelling causes its increase and the erosion, which can only take place after complete hydration of outer layer, causes its decrease [29,42]. However, there is no universal drug release mechanism that can be valid for all systems containing polymer [43].

The mobility of the polymer chains is affected by the dissolution medium composition which should be thermodynamically compatible with the matrix [44,45]. The polymer can then undergo the relaxation process due to the decrease in the glass transition temperature, its chains become more flexible giving volume expansion which is followed by swelling of the system [44,45,46]. It can be expected that hydrophilic polymer may lead to improving of wetting properties leading to the enhancing the diffusion of dissolution medium to the solid powder. However, this assumption can be also greatly influenced by the intermolecular interactions and therefore, also by the arrangement of drug-polymeric carrier structures in the solid dispersions resulting in the changes of functional groups orientation. For this reason, some polar functional groups can be then available or vice versa unavailable for the interaction with dissolution medium during absorption [47].

## 2. Materials and Methods 

### 2.1. Materials

Tadalafil (TAD) was obtained from Zentiva Group, a.s. (Prague, Czech Republic). Kollidon^®^ 12 PF (K12) and Kollidon^®^ VA 64 (K64) were purchased from BASF Pharma (Ludwigshafen, Germany). Methanol and acetonitrile were of LC/MS grades and were obtained from Fisher Scientific Ltd. (Pardubice, Czech Republic). Methanol for solvent evaporation method, hydrochloric acid, potassium dihydrogenphosphate (KDP) and sodium hydroxide were purchased from Penta s.r.o. (Prague, Czech Republic). Sodium dodecyl sulfate (SDS) was obtained from Fluka (Buchs, Germany).

### 2.2. Preparation of Physical Mixtures

Physical mixtures (PMs) were prepared by mixing TAD and K12 or K64 (5% of TAD in PM) with a conventional tumbling Turbula mixer (T2F model, W.A. Bachofen, Basel, Switzerland) for 1 h at 50 rpm.

### 2.3. Preparation of Solid Dispersions

Solid dispersions (SDs) were prepared by various methods. The methods are described in more detail below.

#### 2.3.1. Solvent Evaporation Method (SE)

To prepare for amorphous SDs of TAD in one of the polymeric carriers at 5% drug loading, 2 g of PM was dissolved in 50 or 100 mL of methanol. The drug/polymer solution was then evaporated at 35–40 °C and at 150 rpm under vacuum (150 mbar) with rotary vacuum evaporator LABOROTA 4000 Heidolph (Maneko, Prague, Czech Republic). The thin cast film was collected in a flask and subsequently dried at 40 °C in an oven at least 24 h. The resulting SD was ground using mortar and pestle.

#### 2.3.2. Spray Drying Technique (SPD)

PM (2 g) containing TAD and K12 or K64 was dissolved in methanol (50 mL) to form the solution which was mixed using sonification. After complete dissolution in methanol, the solution was spray dried using the Mini Spray Dryer B-290 (Büchi, Flawil, Switzerland) with an inert nitrogen loop. The aspirator flow was set at 90%, the inlet temperature was kept at 90 °C and the outlet temperature was set at 50 °C. The atomization gas flow was 35% and the pump speed was 3 mL/min.

#### 2.3.3. Hot-Melt Extrusion (HME)

Hot-melt extrusion was performed using universal single screw (L/D ratio 19/25D) PLASTI-CORDER Lab-Station extruder (Brabender Technologie GmbH & Co. KG, Duisburg, Germany) equipped with the heating barrel which is divided into three temperatures zones and a homogenization zone at the end of the screw. The following extrusion temperatures were used for TAD-K12 PM: 140, 150, and 155 °C, and for TAD-K64 PM: 160, 165, and 160 °C. The extruder was manually fed with the 50 g of each PMs. The screw speed was set to 15 rpm for both PMs. The extrudates were then cooled to laboratory temperature on a conveyor belt and milled to fine powder using hammer mill Polymix^TM^ PX-MFC 90 D (Kinematica^TM^, Loughborough, UK) equipped with sieve (mesh size 0.2 mm). The rotational speed was 5000 rpm. The obtained powder was subsequently sieved using a vibratory sieve (AS 200 basic, Retsch, Haan, Germany) for 10 min (amplitude of vibration was 50 mm). The particle size fractions of 125–250 μm (HME125) and 250–425 μm (HME250) were used for further analysis.

### 2.4. X-Ray Powder Diffraction (XRD)

X-ray powder diffraction patterns were determined with D2 PHASER diffractometer (Bruker, Billerica, MA, USA) configured with 1D SSD detector in Bragg-Brentano parafocussing geometry. A Cu Kα radiation was used (wavelength = 1.5416 Å, voltage = 30 kV, current = 10 mA). The samples were analyzed at room temperature over the range of 5°–80° (2*θ*). The time per step was 0.3 s and the increment was 0.020° (2*θ*). The obtained data were evaluated using X´Pert High Score Plus program with the PDF2 database (Malvern Panalytical Ltd., Royston, UK).

### 2.5. Scanning Electron Microscopy (SEM)

To observe the surface morphology of all components, their PMs and SDs, scanning electron microscopy studies were performed using TESCAN VEGA 3 LMU (Tescan, Brno, Czech Republic). A graphite double-sided adhesive tape was covered by samples and then the samples were made conductive by sputter-coating with 5 nm of gold using rotary-pumped sputter coater (Quorum Q150R ES, Quorum Technologies Ltd., Laughton, UK). The images were captured at magnifications factors of 500.

### 2.6. Fourier Transform-Infrared (FT-IR) Spectroscopy

FT-IR spectra were recorded with the NICOLET iS FT-IR spectrometer (Thermo Fisher Scientific Waltham, MA, USA). ATR module was used to measure FT-IR spectra. Each spectrum was measured using spectral resolution of 2 cm^−1^ in 4000–400 cm^−1^ range.

### 2.7. Stability Studies

SDs were exposed to the temperature of 40 °C and to the relative humidity (RH) of 75% in the humidity chamber, model HCP 108 (Verkon s.r.o., Prague, Czech Republic) for twelve months. The samples were stored in powder form and in the case of SDs prepared by hot-melt extrusion also in extrudate form.

### 2.8. Tadalafil Solubility Test

The solubility test was performed in a paddle dissolution apparatus Sotax AT7 Smart (USP 2; Sotax, Basel, Switzerland). Solubility testing was measured for powder TAD (2.5 mg) in various dissolution media (1000 mL), i.e., phosphate buffer (pH 7.2 and containing 6.8 g KDP and 0.9 g NaOH), phosphate buffer + SDS (pH 7.2 and containing 6.8 g KDP, 0.9 g NaOH and 5 g SDS), 0.1 M hydrochloric acid, and 0.1 M hydrochloric acid + SDS (containing 5 g SDS), which were heated to 37 °C. The rotational speed was set to 150 rpm. The samples of 5 mL were taken in following times: 2, 5, 10, 20, 30, and 45 min, and the concentration of TAD was measured using HPLC. Each experiment was performed twice and the mean values of TAD concentration with their standard deviations were calculated.

### 2.9. Dissolution Studies

The release of TAD from PMs and corresponding SDs was studied by different dissolution techniques which are described in more detail below. Each dissolution experiment was performed twice and the mean values of TAD release amount with their standard deviations were calculated.

#### 2.9.1. Flow-Through Cell Method

The dissolution studies for SDs and PMs were carried out in the USP 4 compliant flow-through cell apparatus Sotax CE1 (Sotax, Basel, Switzerland) with piston pump Sotax CY1 (Sotax, Basel, Switzerland). The dissolution flow-through cell for powders and granules having the diameter of 12 mm and a height of 32 mm was employed to study the different samples in all experiments. Each experiment was conducted using the cell in an open-loop system with fresh dissolution medium from the reservoir continuously passing through the cell. The open-loop system was selected for samples due to the low solubility of TAD and requirement of high volume of solvent. The dissolution medium was pumped through the cell with a piston pump. One glass bead of 5 mm diameter was placed in the apex of the cone to protect the inlet tube according to the manufacturer´s instruction. The conical part of the cell was filled with glass beads of 1 mm diameter to ensure laminar flow profile. Two sieves and the amount of powder (approximately 0.02 g) to be studied were placed on the top of the layer of small beads. One sieve was also placed above the powder. The cell was closed with the filter assembly (containing glass microfiber filter GF/D, Whatman^®^, Fisher Scientific Ltd., Pardubice, Czech Republic) to prevent undissolved material from escaping.

The dissolution medium and the apparatus were placed into the water bath and heated to 37 °C. 0.1M hydrochloric acid with 0.5% SDS (*w*/*v*) was used as dissolution medium. The dissolution medium was then degassed. The flow rate of dissolution medium through the cell was set to 23 mL/min. The samples were collected into the beakers for the following times: 10, 20, 30, 40, 50, 60, 70, 80, 90, 100, 110, 120, 150, 180, 240, 360, 480, 600, and 900 s. Aliquots of 1 mL were then withdrawn for the HPLC analysis.

The TAD concentration in the dissolution medium was calculated using a calibration curve which was obtained from the samples of known concentration and the dissolution profiles were normalized to 100% TAD released at the end of the experiment.

The obtained concentration *c* was used for calculation of the dissolution rate r(t) according to Equation (1):

(1) $$r\left(t\right)=\frac{c\left(t\right)\cdot Q}{V_{C}},$$

where Q is the flow rate of dissolution medium (mL/min) and $V_{C}$ is cell volume (dm^3^).

The dissolution rate can be then used for the calculation of the amount of dissolved TAD Δm (Equation (2)):

(2) $$\Delta m=Q\int_{0}^{t}c\left(t\right)dt,$$

#### 2.9.2. Apparent Intrinsic Dissolution Rate (Wood´s Apparatus)

The apparent intrinsic dissolution rate (AIDR) technique was introduced in our previous study [48] as an extension to intrinsic dissolution rate (IDR) to measure matrix related dissolution effects of drug formulation rather than pure drug substance. Dissolution testing of compressed formulations was performed in dissolution apparatus Sotax AT7 Smart (Sotax, Basel, Switzerland) equipped by a rotating disc device. For rotating disk dissolution rate, 200 mg of the PMs or SDs were compressed by the compaction force of about 2.5 tons and using standard die of 10 mm diameter for 1 min using laboratory manual hydraulic press (Specac, Orpington, UK). The die was then attached to the rotor shaft and was immersed into 1000 mL of the dissolution medium. The rotational speed was 150 rpm. The tablets were dissolved in 0.1M hydrochloric acid with 0.5% SDS (*w*/*v*) which was maintained at 37 °C. The samples of 1 mL were taken in following times: 2, 4, 5, 6, 8, 10, 15, 20, 25, and 30 min. The concentration of TAD was measured by HPLC. The apparent intrinsic dissolution rate can be then calculated according to Equation (3):

(3) $$IDR=\frac{V}{A}\frac{dc}{dt},$$

where V is volume of dissolution medium (L) and A is area of exposed tablet surface (cm^2^), c is concentration of dissolved TAD (mg/L) and t is time (s). Unlike IDR measurement, the dissolution profile can be non-linear and thus AIDR is time-sensitive. For example, IDR at *t* = 0 can be used as a measure of drug dissolution unhindered by formed polymer layer.

### 2.10. HPLC

The concentration of TAD in the samples was determined by LC Prominence system (Shimadzu, Kyoto, Japan) equipped with a PDA detector without further dilution and a Kinetex^®^, 5 μm, C18, 100 Å column (Phenomenex^®^, Prague, Czech Republic). Separation was performed with 20 μL injection volume. The flow rate of mobile phase was 1 mL/min and oven temperature was 30 °C. Tadalafil was monitored at 284 nm.

A solution of methanol, acetonitrile and distilled water in ratio 45:40:15 (*v*/*v*/*v*) was used as the mobile phase. The mobile phase was then degassed prior to analysis.

### 2.11. Differential Scanning Calorimetry (DSC)

DSC was performed on the pure TAD, polymers, PMs and SDs. DSC analyses were examined using a differential scanning calorimeter DSC 131 (Setaram, Caluire, France) under nitrogen atmosphere in the temperature range of 25–350 °C. The temperature program was set to a linear increase of temperature at a heating rate of 10 °C/min.

## 3. Results and Discussion

### 3.1. Characterization of Solid Dispersions

It has been documented that the physicochemical properties of amorphous forms can be largely dependent on methods for their production, as well [16].

#### 3.1.1. SEM

**Figure 1:**
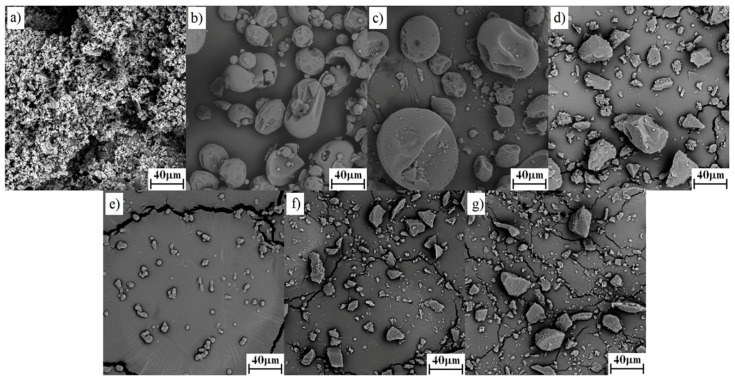
Morphology images of TAD, K12, their PM and SDs: (**a**) pure TAD, (**b**) pure K12, (**c**) TAD-K12 PM, (**d**) TAD-K12 SD (SE), (**e**) TAD-K12 SD (SPD), (**f**) TAD-K12 SD (HME125) and (**g**) TAD-K12 SD (HME250).

**Figure 2:**
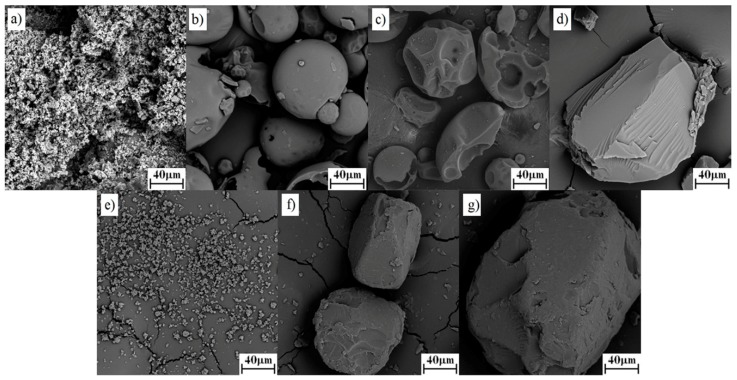
Morphology images of TAD, K64, their PM and SDs: (**a**) pure TAD, (**b**) pure K64, (**c**) TAD-K64 PM, (**d**) TAD-K64 SD (SE), (**e**) TAD-K64 SD (SPD), (**f**) TAD-K64 SD (HME125) and (**g**) TAD-K64 SD (HME250).

For morphological characterization, SEM analysis was performed on the pure drug and both polymer, their PMs and SDs. The SEM images for pure drug, polymers, PMs and SDs of both polymers are shown in Figure 1 and Figure 2. Pure drug images (Figure 1a or Figure 2a) showed fine crystalline powder of irregular shapes and uniform sizes, whereas the images of polymers (Figure 1b for K12 and Figure 2b for K64) showed a spherical particle shape with holes. From the SEM of both pure polymers, it is clear that the K64 contained larger particles in comparison to K12. In the PMs (Figure 1c and Figure 2c), the small particle size TAD was adsorbed on the surface of polymers. The images of SDs of TAD with K12 or K64 did not show any crystalline materials (Figure 1d–g and Figure 2d–g) and the SDs appeared as particles of irregular shape. As can be seen, the type of carrier and different methods strongly affected the morphology of the SDs. Further, only spray drying led to the formation of round and small particles.

#### 3.1.2. FT-IR

**Figure 3:**
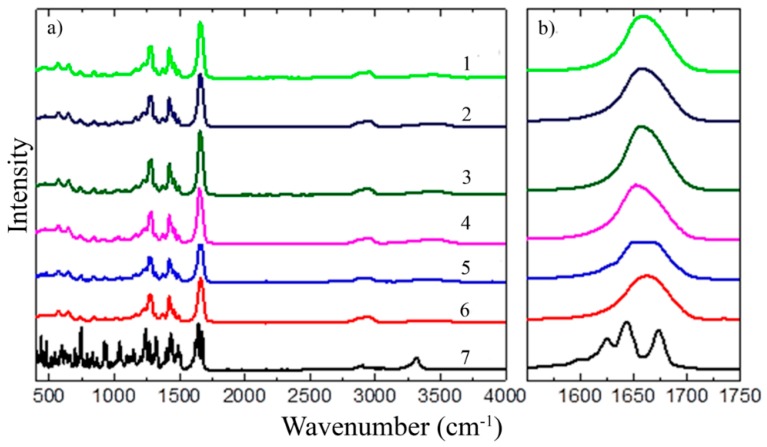
FT-IR spectra of pure TAD, K12, their PM and SDs: (**a**) **1**—TAD-K12 SD (HME250), **2**—TAD-K12 SD (HME125), **3**—TAD-K12 SD (SPD), **4**—TAD-K12 SD (SE), **5**—TAD-K12 PM, **6**—pure K12, **7**—pure TAD and (**b**) their detail spectra at 1600–1750 cm^−1^.

**Figure 4:**
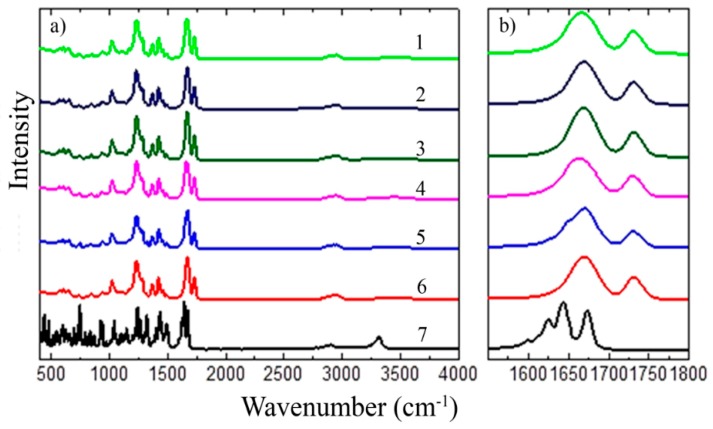
FT-IR spectra of pure TAD, K64, their PM and SDs: (**a**) **1**—TAD-K64 SD (HME250), **2**—TAD-K64 SD (HME125), **3**—TAD-K64 SD (SPD), **4**—TAD-K64 SD (SE), **5**—TAD-K64 PM, **6**—pure K64, **7**—pure TAD and (**b**) their detail spectra at 1600–1800 cm^−1^.

FT-IR spectra were measured to study TAD-polymer matrix interactions. In the Figure 3a and Figure 4a, the FT-IR spectra of pure TAD, K12 and K64, their PMs and SDs at 500–4000 cm^−1^ are shown. The Figure 3b and Figure 4b also show the detail spectra at 1600–1750 cm^−1^ or 1600–1800 cm^−1^, respectively.

TAD showed the signal of the stretching vibration of secondary amine group (3321 cm^−1^), the signal of aliphatic methyl group (2904 cm^−1^), the dual signal of carbonyl groups (1673 cm^−1^), the signal of aromatic C=C bending (1649 cm^−1^) and the signal of tertiary amine group (1270–1285 cm^−1^). K12 had the carbonyl stretching band that is at 1661 cm^−1^. The FT-IR spectra of pure K64 had aliphatic alkyl C–H stretch that is in mutual association at 2937 cm^−1^, the signal of vinyl acetate (1730 cm^−1^) and the signal of carbonyl group (1669 cm^−1^). The spectra of all physical mixtures displayed tadalafil and polymer peaks with decreased peak intensity but with no shifting of the peaks. FT-IR spectra of all SDs showed the presence of some TAD peaks with decreased intensity as was described e.g., in [8]. Shifts of characteristic bands are not visible in the spectra, but only changes in their intensity or changes in the sub-band intensity of multi-component bands, as confirmed by a detailed analysis of the second spectra derivatives. All other tadalafil peaks were smoothened, indicating a strong physical intermolecular interaction of TAD with polymers. The decreasing intensity of the carbonyl group was observed for TAD-K12 PM and its SDs suggesting the presence of hydrogen bonds between the TAD secondary amine group and K12 carbonyl groups. Molecular interaction with polymer, predominantly through hydrogen bonding which was also described in [19], leads to better miscibility of TAD in the polymer matrix and the formation of amorphous SDs, as confirmed by XRD results. The interactions were stronger in the TAD-K12 SDs in comparison to TAD-K12 PM. Stronger drug-polymer interactions could be generated for example due to the melt extrusion [2].

The changes in the intensity of the carbonyl group were also observed in the TAD-K64 PM and its SDs. Even the pure polymer has other sub peaks visible on the deconvolution by curve fitting of its characteristic absorbing bands for carbonyl (1662 cm^−1^) and for vinyl acetate (1730 cm^−1^). These are vibrations of different types of groups, in which the carbonyl may be influenced by either two other carbonyls, optionally one or two vinyl acetate group in the neighboring position, or it may be a terminal group where the bond energy and its vibration depends on the neighboring monomer unit. After deconvolution of main bands, changes in the intensity of some peaks can be observed. In the case of a TAD-K64 PM, the vinylacetate group is also weakened. As a result of the different preparation of SDs, different relaxation of the polymer and its recombination occurs and leads to the change in the structural arrangement exhibiting different carbonyl group orientations. This is reflected in the change in the intensity ratio between the deconvoluted peaks, which is most noticeable between the TAD-K64 SD (SE) and TAD-K64 SD (HME125) spectra. Each of these mixtures was prepared at a completely different temperature and manifested in, for example, different dissolution behavior (see below). However, the interaction between the polymer and TAD can be observed with other vibrations than with the NH-vibration. Several different types of structures can be observed relative to each other for the vibrations of –CH– in the region 3000–2800 cm^−1^ range, or 2925 cm^−1^ corresponding to –CH_2_ groups. CH deformation oscillations in the area of 1460 cm^−1^ again show the differences between the SDs and the PM when the PM exhibits more pronounced peaks, whereas, for example, in the TAD-K64 SD (SE), the absorption bands are more diffuse and overlap each other. However, no additional peak was observed in any binary system indicating absence of any another chemical interaction between TAD and polymer [5,49].

#### 3.1.3. XRD

**Figure 5:**
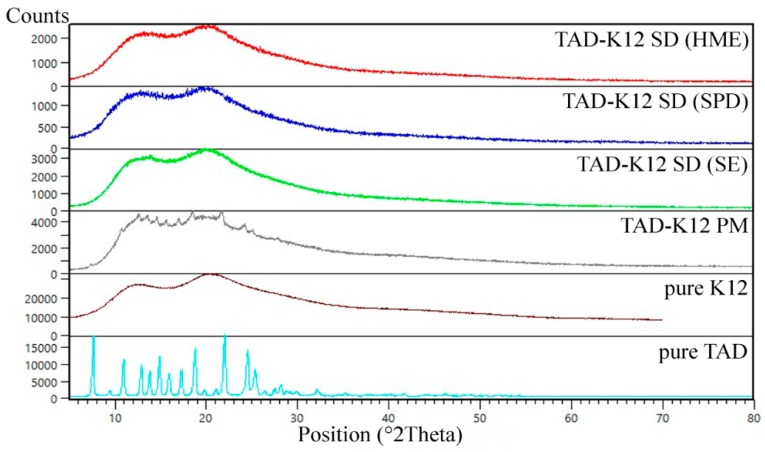
X-ray diffraction patterns of TAD, K12, their PM and SDs.

**Figure 6:**
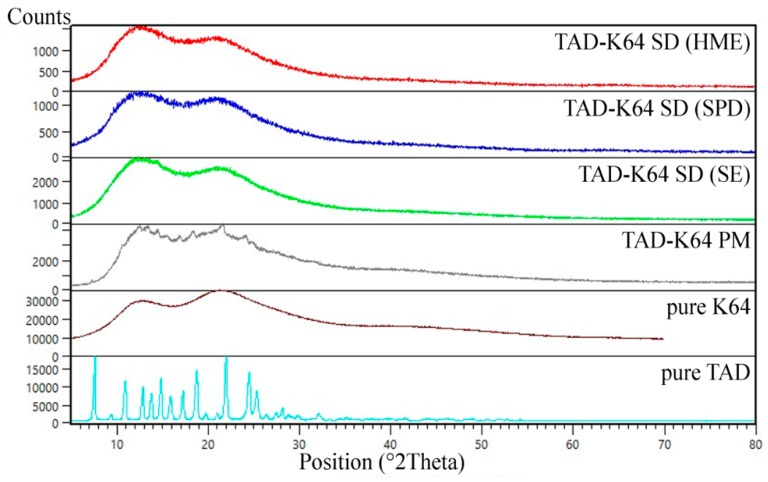
X-ray diffraction patterns of TAD, K64, their PM and SDs.

The powder X-ray diffraction patterns of pure TAD, K12 and K64, their PMs and SDs prepared by various method are shown in Figure 5 and Figure 6, respectively. The diffraction pattern of the TAD had sharp intensive peaks throughout its pattern suggesting that it is crystalline in nature [15]. On the contrary, the polymeric carriers showed broad amorphous bands and no sharp diffraction peaks suggesting that the Kollidons were in amorphous state. The diffraction patterns of PMs showed sharp peaks corresponding to crystalline TAD and therefore, we can assume that crystalline TAD can be detected in its low concentration in all binary mixtures. The TAD peaks then disappeared in all SDs loaded with 5% TAD and therefore, their amorphous forms were confirmed. All extrudates were transparent, that means the dissolution of TAD in amorphous polymers.

#### 3.1.4. DSC Analysis

**Figure A1:**
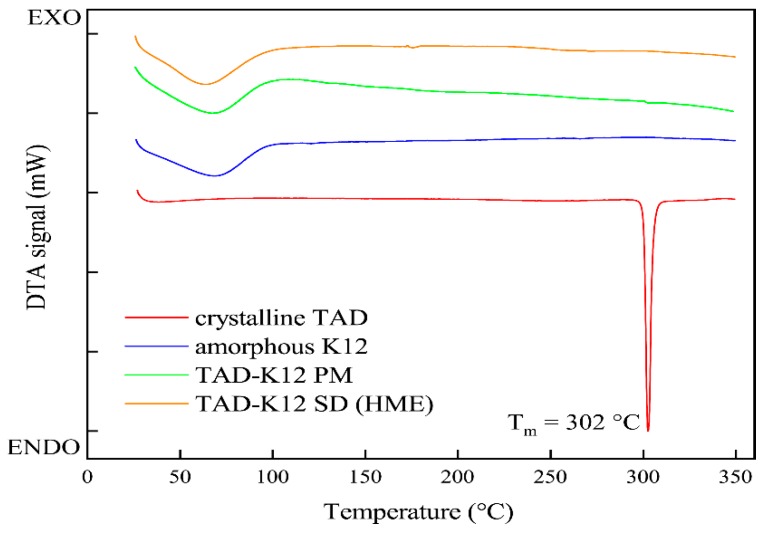
DSC thermograms of (from bottom to top) crystalline TAD, amorphous K12, TAD-K12 PM, and TAD-K12 SD (HME).

**Figure A2:**
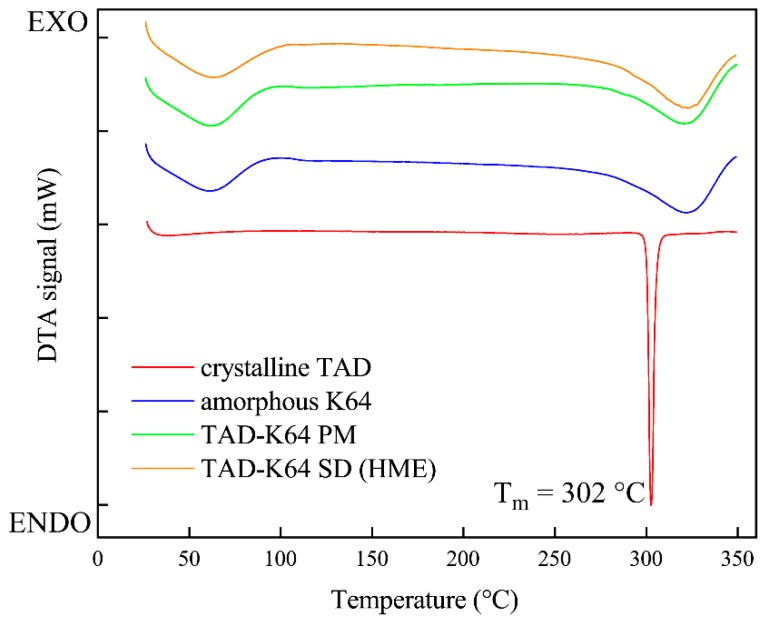
DSC thermograms of (from bottom to top) crystalline TAD, amorphous K64, TAD-K64 PM, and TAD-K164 SD (HME).

Figure A1 and Figure A2 (see Appendix A) present DSC thermograms for crystalline TAD, amorphous K12 or K64, their PMs and SDs prepared by HME as an example. DSC thermograms of both SDs reveal the absence of any melting peak which means that these results suggest amorphous characteristics of SDs. In other words, it indicates the absence of crystalline trace of TAD in SDs. Besides, a broad endotherm ranging from 25 to 100 °C was observed in thermograms of pure K12, K64, both PMs and SDs which can be attributed to the water loss from the hygroscopic polymer upon heating. Figure A1 or Figure A2 also show a sharp melting endotherm at 302 °C corresponding to crystalline TAD. Moreover, no significant glass transition temperature was seen in thermograms. In order to detect the T_g_, it is necessary to decrease heating rate. However, both Kollidons tend to degrade at lower heating rates. Figure A2 also show broad endotherms ranging from 300 to 350 °C. The degradation temperature of K64 is 230 °C. Based on this, broad endotherms can be attributed to some thermal event corresponding to the decomposed polymer.

### 3.2. Physical Stability of Solid Dispersions

**Figure A3:**
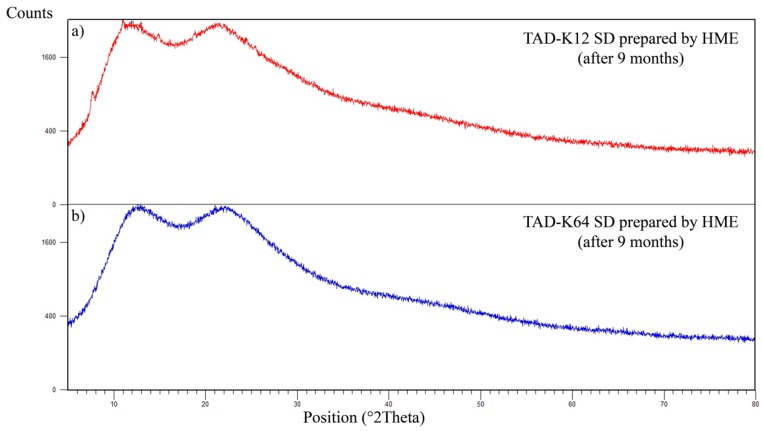
X-ray diffraction patterns of extruded SDs after 9 months: (**a**) TAD-K12 and (**b**) TAD-K64.

The samples were stored in powder form and in the case of SDs prepared by HME also in extruded form. Stability testing at 40 °C and 75% RH for 12 months revealed that extrudates of both Kollidons to be amorphous for nine months and their powder forms to be amorphous for 6 months which was confirmed using XRD analysis as shown Figure A3a,b in Appendix A. Figure A3 shows results from XRD only for extrudates because X-ray diffraction patterns were identical also for powder forms prepared by various methods. Therefore, no crystalline form of the TAD was noticed after this period. The presence of hydrogen bonds between the drug and the polymer can lead to their better miscibility and effectively improves the physical stability of the SDs and therefore, they are essential [2,50]. Hydrogen bonds stiffen the structure and thus, hinder the diffusion of molecules [3]. In our case, the hydrogen bonds between the components were observed. SDs of both polymers in powder form changed their character after 6 months and for this reason, the sticky viscous solution was formed. Since the glass transition temperature is dependent on relative humidity [51], its decline can be expected leading to the glass solutions. In the case of SDs in extruded form, this phenomenon was observed after 9 months. However, all these viscous solutions were still transparent which could mean that TAD maintain its amorphous form.

### 3.3. Dissolution Tests of TAD Solid Dispersions

It has been reported that amorphous solid dispersion formulations require the use of many excipients for the optimal design, especially to maintain supersaturation and improve physical stability or to ensure shelf-life stability and better absorption during intestinal transit [52]. Choi et al. prepared tadalafil solid dispersion coupled with the incorporation of an acidifier and solubilizer. They found that both tartaric acid increasing wettability and Soluplus^®^ improving solubility contribute to the dissolution rate. Their optimal formulation also contained Aerosil 200 to ensure better flow properties and drug stability [12]. In another study, Choi et al. used malic acid and meglumine at lower contents in the preparation of tadalafil solid dispersion to improve drug solubility and dissolution rate, and Aerosil 200 to improve drug dispersibility [19]. Choi et al. also investigated the effect of various weak acids and bases on tadalafil solid dispersion formulation. Their results indicate that only meglumine significantly improve the apparent drug solubility and dissolution [20]. Obeidat et al. evaluated a mixture of surfactants (Tween 80 and Span) as stabilizers [13]. However, we focused primarily on the effect of different structural polymers and methods of solid dispersion preparation on the dissolution mechanism and release kinetics of tadalafil from amorphous solid dispersions. These properties strongly depend upon the nature of all components, but the dissolution rate of the drug is mainly affected by the aforementioned factors (see Introduction) which are associated with the polymer carrier. Therefore, the drug to polymer carrier ratio was fixed at 5% drug loading in order to maximize the effect of polymer solubilisation and probability complete amorphous form. The pure TAD drug and PMs thereof with the carrier materials were used as references.

#### 3.3.1. Solubility Testing

**Figure 7:**
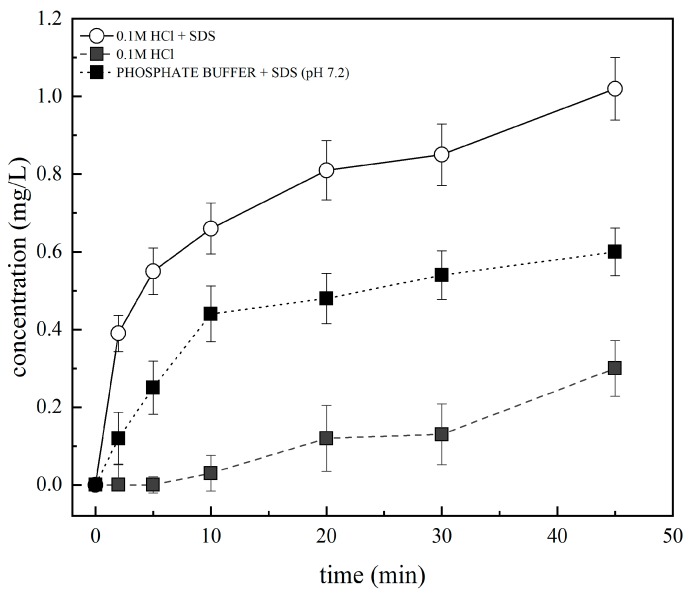
Solubility testing of TAD in various dissolution media.

Prior to the dissolution tests with the polymer, the solubility studies of pure TAD (as was received) were compared in various dissolution media, as shown in Figure 7.

TAD is classified as class IV in the Biopharmaceutical Classification System and is practically insoluble in water (2 μg/mL) [53]. It was found that the solubility of TAD was maximum in 0.1 M hydrochloric acid + SDS. On the other hand, the solubility of TAD was minimum in 0.1 M hydrochloric acid. Moreover, the results of TAD solubility in phosphate buffer (pH 7.2) + SDS were affected by the ion exchange between phosphate buffer and SDS. For this reason, these data were not included in the evaluation. Dissolution tests were then performed in 0.1M hydrochloric acid + SDS. The addition of SDS to the dissolution medium can have an impact on the magnitude of drug concentrations, but the relative order of the release profiles should not be changed, particularly at low drug concentration [16].

#### 3.3.2. Assessment of the Erosion-Diffusion Mechanism of TAD Release Using Wood´s Apparatus (AIDR Measurements)

**Figure 8:**
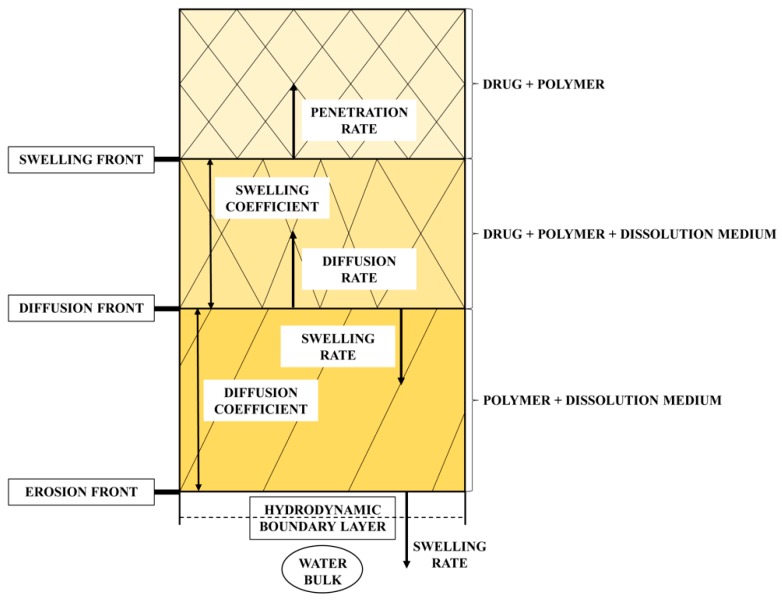
Scheme of front position during dissolution.

A general mathematical model describing the dissolution behavior was proposed e.g., in [48,54,55]. Moreover, AIDR was described in [48]. However, there is still no model currently available to describe the solubility and dissolution behavior of amorphous SDs formulated with hydrophilic polymers [56]. Drug release from hydrophilic matrices is generally considered to be governed by the complex interactions between dissolution, diffusion and erosion mechanisms [57]. Pharmacopoeia defines the intrinsic dissolution rate for pure API (active pharmaceutical ingredient). Therefore, if some excipient is added to this drug, we can then observe whether the release rate is accelerated or retarded (see Figure 8).

Such dissolution tests can be performed using Wood´s apparatus [1]. The transport phenomena can then only be allowed in axial direction. It gives the advantages of exposing a constant surface area of the formulation to the dissolution medium, and therefore it eliminates the influence of different surface area of powders by their compaction [26].

**Figure 9:**
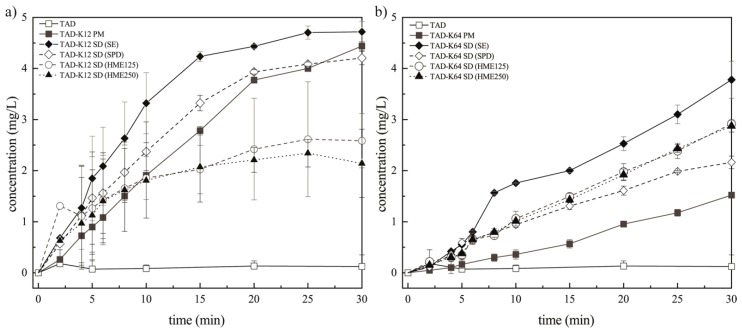
Release profiles of TAD from PMs and SDs prepared by different methods measured as apparent *IDR* using Wood´s apparatus: (**a**) K12 groups and (**b**) K64 groups.

Prior to dissolution tests with the polymer, the release rates of pure TAD were performed as reference. The release profiles of TAD from PMs and SDs with various polymers prepared by different methods are shown in Figure 9.

Table 1 and Table 2 summarize the values of AIDR for all tested samples. The slope of the initial linear part of a release profile was used as AIDR for samples which exhibited curved profiles (namely up to the 10th minute for PM and SDs containing K12).

The release profile from SDs obviously varied depending on the methods of preparation but there is notable improvement in release rate and the quantity of released TAD for all SDs over pure TAD. The release rates of TAD were found to be significantly different from each specific method and polymer. Figure 9 reveals that there was noticeable influence of polymers and preparation methods on TAD dissolution rate. It is also clear that the pure TAD had the lowest dissolution rate. As can be seen in Figure 9a, the presence of hydrophilic soluble K12 caused faster medium penetration, faster release and faster polymer erosion in comparison to pure TAD. The initial TAD release rate is faster from PM and SDs than pure TAD release rate in the first 15 min followed by a second stage with a slower almost constant drug release rate. A subsequent slowdown in the dissolution was not caused by saturation of the solution with TAD but might have been the effect of progressive swelling of polymers and hindered diffusion of TAD molecules from polymer, as was described e.g., in [3]. During this diffusion period, the thickness of the viscous gel layer on the tablet surface increased over time, leading to longer diffusion path for TAD into the bulk dissolution medium. Consequently, the release of TAD was then limited by water penetration to the tablets as well as drug diffusion through the gel layer. That suggests this type of release is only due to the combination of erosion and diffusion mechanisms.

In general, up to three phases can be observed in the AIDR profiles in Figure 9. The initial AIDR increase for SDs over that of the pure TAD is caused by improving TAD wettability by surrounding hydrophilic carrier, reducing TAD particle size effectively to single molecules and reducing its crystallinity. This is also facilitated by enhanced water penetration into the matrix and the polymer swelling. As the swelling and the diffusion fronts travel deeper into the matrix, the distance between the matrix surface (erosion front) and the diffusion front increase, thus increasing the diffusion path length, leading to reduced AIDR over time, which is represented by the second (transient) phase of gradually decreasing slope of the release profile for some formulations, especially those using the K12 polymer. This AIDR reduction means also slower progress of the diffusion front. Once the diffusion front progress become equal to that of the erosion front, the thickness of the diffusion layer and length of the diffusion path of TAD approaches its steady state, which results in the third phase of steady release at slower rate than in the initial phase. This phase is displayed for the PM and SD (prepared by SE or SPD) formulations using the K12 polymer at dissolution times over approximately 20 min. Some formulations may never reach the third phase (e.g., HME formulations using K12), which means the progress of the erosion front is negligible. Yet other formulations may maintain their initial AIDR (e.g., K64 formulations) without entering the transient second phase, which means the erosion front progress at the same rate as the diffusion front from the very start of the experiment and no significant diffusion barrier is developed. The fastest TAD release, as well as its largest amount released, was observed in SD prepared by SE. The different morphology of particles (obtained SEM) resulted in differences in disk intrinsic dissolution rate or also in medium apparent solubility (see below). This is probably because SD particles contain irregular fracture edges (Figure 1d) leading to increase in surface area and therefore, to faster TAD release. Furthermore, it is shown that TAD release rate of SD prepared by SPD is almost comparable to the corresponding PM. The incorporation or encapsulation of drug into the matrices during spray drying can affect the drug release rate [2,58]. The swellability has a significant effect on the release kinetics of an incorporated drug [43]. Therefore, TAD was probably encapsulated by K12 during spray drying and TAD release was then slower. In the case of SD prepared by HME, TAD release is the slowest in comparison to other SDs or PM containing K12. TAD dissolution rates from these extrudates were considerably greater in comparison with the pure TAD and PM at the initial phase, even though they were observed to decrease over the duration of the study. Their dissolution profiles overtake that of the PM at the beginning of the experiment (approximately during 10 min). This is probably because TAD is better wetted in the both sieve fractions of extrudates. Since their dissolution profiles overlap, they were no noteworthy differences in the porosity between both extrudates as was described e.g., in [15]. Subsequently, its release is probably retarded by the gel layer and therefore, diffusion mechanism predominated.

It is clear from Figure 9b that polymer dissolution influences the TAD release profile significantly. Drug release decreased if polymer molecular weight increased [59] and for this reason, polymer erosion rate increased with the decrease of polymer molecular weight. K12 has a lower molecular weight (2500 g/mol) in comparison to K64 (45,000 g/mol), wherein greater the molecular weight, higher the viscosity and slower the release can be observed. During SDs preparation, solid dispersions containing K64 was of high viscosity, especially in HME. Consequently, the pathways to be overcome by the TAD to be released were very much shortened in the case of the systems containing K12. For this reason, the TAD releases were influenced by the polymer molecular weight because at high molecular weights, the polymer was also more entangled. The initial polymer dissolution rate is usually zero until the entanglement strength is reduced by the increased penetrant concentration and the polymer dissolves because of chain disentanglement [60,61]. Therefore, the polymeric segments of K12 were not so entangled and for this reason, the thickness of the viscous layer on the tablet surface was weaker in comparison to tablets containing K64. The gel strength corresponds actually to resistance to dissolution media penetration [62]. The tablets containing K64 displayed a totally linear release profile, whereas the tablets containing K12 showed a non-linear release (Figure 9). Therefore, the tablets containing K12 seems to reach the plateau where no more TAD is dissolved after 15 min, whereas the TAD still continues to be released from the tablets containing K64 even after 30 min of the experiment as explained by the different erosion rate above. The linear release providing that the releasing area is kept constant was also observed in [39] when the polymer was sufficiently soluble and therefore, the gel layer thickness remained constant because the fronts in the matrix moved in a synchronized way. It can be caused by the chemical structure of the Kollidons. Figure 9b shows the swelling of the tablets (upon complete hydration approximately during 5 min) and gradual erosion of K64. It can be attributed to the fact that K64 contains lipophilic vinyl acetate which is insoluble in the aqueous medium and hydrophilic vinylpyrrolidone which can form the channels in the tablets. This phenomenon was described in [29] for Kollidon^®^ SR. Therefore, this monomer allows better capture and penetration of water by the tablets and hence a greater viscosity of the gel. Medium then penetrates into the free spaces on the surface between the polymer chains. TAD can diffuse slowly through these channels which is reflected in its slower release rates in comparison to the tablets containing K12. However, the release rate would be controlled solely by mechanisms of erosion due to higher AIDR values in comparison to pure TAD.

Dissolution from both PMs showed also improvement in the release rate of TAD. However, the fastest TAD release, as well as its largest amount released, was also observed in SD prepared by SE as compared with that of solid dispersions and pure form of TAD. This is probably because SD particles also contain irregular fracture edges (Figure 2d) leading to increase in surface area and therefore, to faster TAD release. Furthermore, it is also shown that the TAD release rate of SD prepared by SPD was retarded in comparison to other SDs. TAD was probably also encapsulated by K64 during SPD. TAD release is then slower, although, the very small SD particles were obtained by this method of preparation (Figure 2e). There was not significant difference between the release rates of SDs sieve fractions prepared by HME and their profiles were practically superimposed. Therefore, the utilization of the same compression conditions for different SDs can paradoxically lead to varying degree of compaction and is crucial in dissolution, as was described in [16]. However, best solid dispersion form of TAD was prepared by solvent evaporation using both polymers. Slower and lower TAD release from all extrudates might also be due to the property of the polymer to undergo thermoreversible gelling at high concentration after the melting process and subsequent cooling during the formulation of SDs [5].

#### 3.3.3. Effect of the Preparation Method on the Dissolution Behavior of TAD from Solid Dispersions

**Figure 10:**
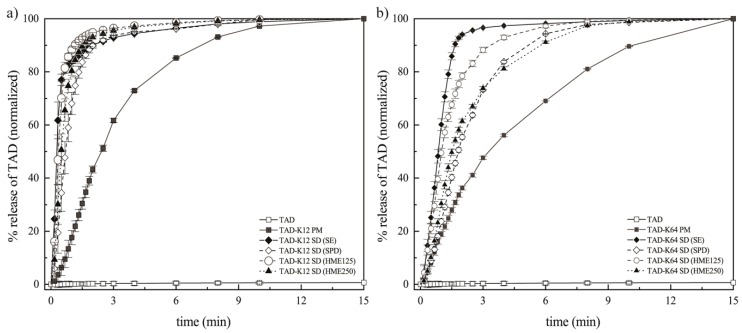
Release profiles of TAD from PMs and SDs prepared by different methods: (**a**) K12 groups and (**b**) K64 groups.

Flow-through cell apparatus for powders was used to describe the dissolution behavior of SDs. The dissolution profiles from the SDs containing K12 or K64 obviously varied depending on the methods of preparation and polymer type. Figure 10 represents normalized data. The normalization was used to more easily compare data from different SDs because possibly non-homogeneity of some samples (especially in the case of TAD-K12 SD HME125 or HME250) were observed. The original data are then shown below (namely in Figure 13). Figure 10a,b shows that the powders containing K12 allow a faster gradual release of TAD from K12 in comparison to powders containing K64.

SDs containing K12 also show similar dissolution profiles that reach the identical asymptotic amounts of TAD in the medium. After the initial dissolution times, most of the TAD released at comparable release rates except for PM which showed the lowest release rates among all SDs. As shown in Figure 10, the TAD amounts, therefore, are very high in the first 3 min, and then the amounts become slower and are almost constant. The same trend can be observed for SDs containing K64, but the dissolution is slower compared to K12. It can be attributed to enhancement of powders wettability. Molecular dispersion of poorly water-soluble drug is the most desirable type in the theory and practice of amorphous solid dispersions because it is related to the enhancement of the solubility by the weakening of solute-solute interactions. TAD has a very high binding energy between its molecules due to its high melting point (302 °C). For this reason, the reduction in the binding energy allows for the easier passage of molecules from the amorphous solid state to the medium. It is additionally facilitated by detachment of polymer chains and subsequent increase in the contact surface with medium leading to the considerable solubility improvement [3]. Therefore, in the case of SDs containing K64, this could be attributed to the lowered mobility of TAD particles or molecules embedded within the K64 matrix system particularly in the initial phase. From the above AIDR measurements, it appears that although the SDs enhance the TAD release rate, in these dissolution experiments, there was not a significant difference between the dissolution behaviors of the SDs. It may be therefore concluded that flow-through cell data measured on powders emphasize the effects related to the prepared particles of SDs and therefore are relevant to situations corresponding to the first phase of the AIDR experiments. Since the particles are relatively small and no tablet was compressed the swelling and erosion effects are suppressed, while the particle surface effects are more pronounced. It is also obvious that the largest amount of TAD was dissolved in the case of SDs containing K12 or K64 prepared by SE. As mentioned above, their SDs contained irregular fracture edges leading to the increase in surface area (Figure 1d and Figure 2d). On the other hand, the TAD slowest release was observed in the SDs containing K12 or K64 prepared by SPD. This was also commented above. The incorporation of TAD into the K12 or K64 matrix also affects the dissolution behavior. Therefore, SDs prepared by SE showed the highest dissolution rate among them, and both extrudates having particles of 125–250 μm showed the second highest dissolution rate among them.

**Figure 11:**
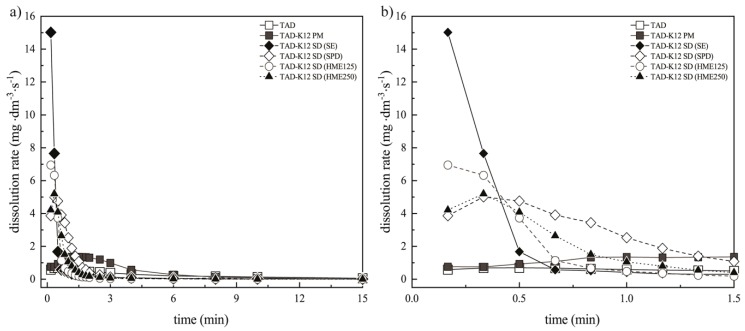
Dissolution rates of TAD from PM and SDs containing K12: (**a**) during 15 min and (**b**) during 1.5 min.

**Figure 12:**
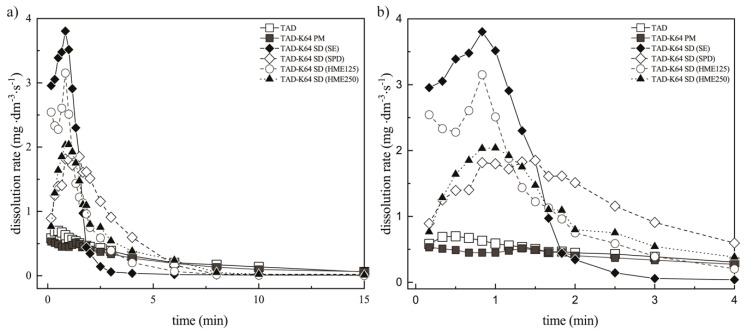
Dissolution rates of TAD from PM and SDs containing K64: (**a**) during 15 min and (**b**) during 4 min.

The results of dissolution studies and the initial dissolution rates during the first 1.5 min (for the systems containing K12) and 4 min (for the systems containing K64) for all SDs and corresponding PMs are depicted in the Figure 11a,b and Figure 12a,b.

The most significant differences between the prepared samples were observed within 1.5 min. For this reason, the Figure 11b and Figure 12b show more detail of the dissolution experiments. The results are represented as data points. From these values, dissolution rates of TAD were always higher from the SDs compared with pure TAD or the PMs. It was also found that the presence of hydrophilic K12 or K64 in the SDs caused faster dissolution rate that was in compliance with the results mentioned above. It was also observed that the SDs containing K12 showed faster dissolution rate compared to K64. The fastest TAD dissolution rate was also measured in the SDs containing K12 or K64 prepared by SE due to the presence of irregular fracture edges. The fast initial rate can be attributed to the hydrogen bonding between TAD and both polymers, which breaks relatively easily during dissolution compared with pure drug [17].

#### 3.3.4. Weibull Dissolution Model

**Figure 13:**
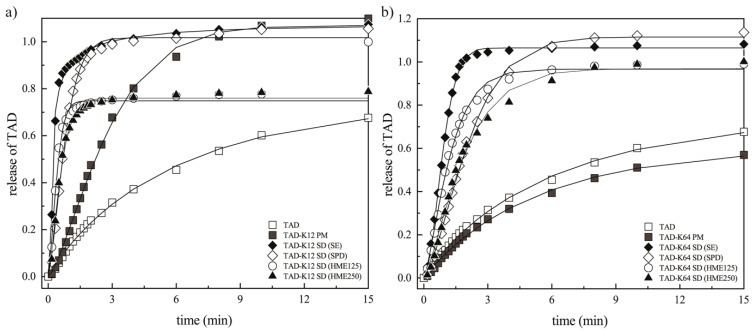
Dissolution profiles of TAD formulations (points) fitted by Weibull model (lines): (**a**) K12 groups and (**b**) K64 groups.

In order to describe the TAD release, the obtained dissolution profiles were fitted to the Weibull model which is adapted to the release process. This kinetic model can be expressed by the Equation (4):

(4) $$A_{t}=A_{\infty }\cdot \left[1-\exp\left(-k\cdot {\left(t-t_{0}\right)}^{b}\right)\right],$$

where $A_{t}$ is the amount of drug released in time t, $A_{\infty }$ is the maximum releasable amount of API, k corresponds to the reciprocal value of time scale of the release process, $t_{0}$ is the location parameter and represents the lag time before the onset of the dissolution (in most case is equal to zero) and b describes the shape of the dissolution curve [63]. When the shape parameter b is equal to one, the Weibull model corresponds to the first order kinetic model and therefore, the parameter k corresponds to the first order release rate constant. ERA software [64] was used for the fitting the Weibull equation to the experimental data. Figure 13 shows TAD release profiles fitted by the Weibull model. As can be seen in Figure 13, the model was in agreement with the dissolution profile and therefore, it was appropriately used. Therefore, the kinetic parameters of the model and their standard deviations (STDs) are summarized in Table 3 and Table 4.

**Figure 14:**
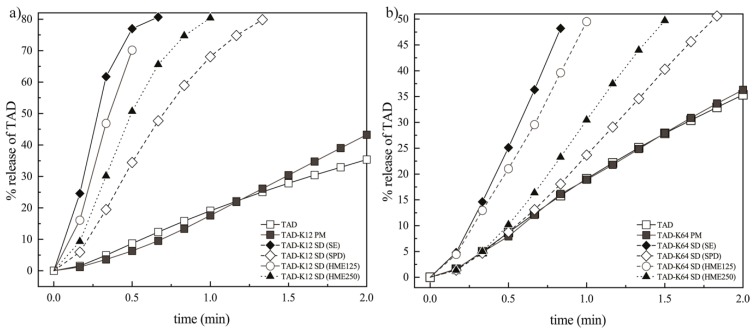
TAD release profile during 2 min: (**a**) K12 groups and (**b**) K64 groups.

Since the parameter k corresponds primarily to the initially release rate, the Figure 14a,b represents TAD release profile with respect to this fact only up to the second minute.

The values of b about 1 were found for pure TAD, SD containing K12 prepared by SPD, PM containing K64 and SD containing K64 prepared by HME (specially for the sieve fraction 125–250 μm) (Table 3 and Table 4). In other case, the release rate is retarded in comparison to the first order kinetic (b < 1), i.e., for SDs containing K12 prepared by SE and HME (both sieve fractions) and SD containing K64 prepared by HME (sieve fraction 250–425 μm). On the other hand, the release rate is accelerated in comparison to the first order kinetics (b > 1), i.e., for PM containing K12 and SDs containing K64 prepared by SE and SPD. These results confirm the fact that the parameter k correlate with the amount of drug released at the beginning of the dissolution experiment.

## 4. Conclusions

In this study, TAD amorphous solid dispersions were successfully obtained using the solvent evaporation method, spray drying technique, as well as hot-melt extrusion. Two grades of polyvinylpyrrolidone (K12 and K64) were used as a polymeric carrier. Therefore, the use of different preparation methods for SDs and polymers having different physicochemical properties can provide greater insights into the importance of various mechanisms of the dissolution process.

SEM revealed significant differences in morphology of all samples which were reflected in the dissolution tests. FT-IR spectra confirmed the interactions between the drug and both Kollidons as well as the presence of hydrogen bonds between the components. These interactions were weaker in the binary mixtures containing K64 compared to K12. The presence of hydrogen bonds between the drug and the polymers improved the physical stability of the SDs in their extruded forms. Therefore, stability studies for nine months confirmed the extrudates to be amorphous. On the contrary, the SDs of both polymers in powder form changed their character due to high relative humidity.

The Wood´s apparatus was found to be suitable for determining the apparent intrinsic dissolution rate and for identification of critical factors affecting the erosion-diffusion mechanism of TAD release. TAD release from compressed SDs containing K12 was controlled by combination of erosion and diffusion mechanisms. The diffusion mechanisms were predominant in the initial phase of experiment and the slow erosion was dissolution-controlling at the second stage. TAD release rate from SDs containing K64 was controlled solely by mechanisms of erosion. The dissolution profiles obtained by Wood´s apparatus were in agreement with dissolution profiles obtained using flow-through cell apparatus. The fastest TAD release, as well as its largest amount released, was observed in SD containing K12 or K64 prepared by solvent evaporation method. This is probably because SD particles contain irregular fracture edges, which was revealed by SEM, leading to increase in surface area and to faster TAD release. In the case of SDs containing K12 or K64 prepared by spray drying, TAD was probably encapsulated by K12 or K64 during spray drying. TAD release was then slower, although, the very small SD particles were obtained by this method of preparation. The effect of polymer molecular weight on the release rate was also observed. K12 has a lower molecular weight in comparison to K64. The TAD releases were then influenced by the polymer molecular weight because at high molecular weights, the polymer was more entangled. For this reason, the SDs containing K12 showed faster dissolution rate compared to K64. Weibull dissolution model was suitably used to describe the TAD release at the beginning of the dissolution experiment as well as to assess whether the release corresponds to the first order kinetics. There was noticeable influence of polymers on TAD solubility. All solid dispersions improved the TAD intrinsic dissolution rate, however, the greatest increase in the TAD dissolution rate was obtained from SD containing K12 prepared solvent evaporation. It can also be concluded that all SDs of TAD showed considerable enhancement in dissolution rate compared to both PMs and the dissolution rate of both PMs was higher compared to the pure TAD. The rapid dissolution of TAD from SDs, especially in the initial phase, may be attributed to its molecular dispersion in polymer carriers.

In order to summarize, the dissolution experiments revealed that the TAD release from the solid dispersion is controlled by different processes in combined mechanism of surface hydrophilization, dissolution, diffusion in swollen matrix, and the matrix erosion, depending not only on the polymer used, but also on the method of preparing the solid dispersion and further processing thereof. All SDs with hydrophilic polymers enhanced the initial dissolution rate of TAD in both the AIDR arrangement and the USP 4 for solid dispersion over the dissolution rate of pure TAD or TAD PMs. While the enhancement occurred for all SDs systematically, it was of different strength for both the different polymers and the different preparation methods. In general, the dissolution was more enhanced by K12 than K64 polymers and the SDs prepared by the solvent evaporation released the TAD faster than SDs prepared by other methods. Those results should be interpreted as the contribution of hydrophilization and TAD dispersion to dissolution. The AIDR experiments also showed the differences in compressed forms prepared from SD particles. Most notably, the two polymers tested exhibited entirely different behavior. While K64 SDs dissolution was not hindered by the diffusion because the polymer matrix erosion controlled the process and the AIDR proceeded at a steady rate for all samples, K12 dispersions exhibited different erosion rates for different preparation methods resulting in very different AIDR profiles and diffusion hindrance, corresponding to different diffusion layers formed by the diffusion-erosion rate equilibrium.

Apparent intrinsic dissolution rate studies and apparent solubility revealed the greatest increase in TAD solubility and significant dissolution rate enhancement for all SDs in comparison with crystalline TAD and its PMs. The proposed SDs based on K12 or K64 showed an interesting potential for improving the oral bioavailability of the poorly water-soluble tadalafil. This information can be also used either to optimize formulation to obtain the desired release profile or provide a better understanding into the mechanism of TAD release from solid dispersions.

## Figures and Tables

**Table 1:** Apparent intrinsic dissolution rate of TAD (K12 polymer matrix).

Sample	AIDR (mg∙min^−1^·cm^−2^)
pure TAD	0.002
TAD-K12 PM	0.380
TAD-K12 SD (SE)	0.662
TAD-K12 SD (SPD)	0.433
TAD-K12 SD (HME125)	0.245
TAD-K12 SD (HME250)	0.279

**Table 2:** Apparent intrinsic dissolution rate of TAD (K64 polymer matrix).

Sample	AIDR (mg∙min^−1^·cm^−2^)
pure TAD	0.002
TAD-K64 PM	0.081
TAD-K64 SD (SE)	0.247
TAD-K64 SD (SPD)	0.156
TAD-K64 SD (HME125)	0.245
TAD-K64 SD (HME250)	0.194

**Table 3:** Kinetic parameters of TAD release (K12 polymer matrix).

Sample	k (s^−b^) ± STD	b (-) ± STD	$t_{0}$ (s) ± STD
pure TAD	0.21 ± 0.04	0.99 ± 0.10	0.00 ± 0.19
TAD-K12 PM	0.29 ± 0.04	1.13 ± 0.08	0.26 ± 0.10
TAD-K12 SD (SE)	1.92 ± 0.06	0.33 ± 0.04	0.17 ± 0.00
TAD-K12 SD (SPD)	1.27 ± 0.87	1.02 ± 1.15	0.13 ± 0.46
TAD-K12 SD (HME125)	2.30 ± 0.36	0.66 ± 0.21	0.15 ± 0.03
TAD-K12 SD (HME250)	1.76 ± 1.15	0.86 ± 1.16	0.14 ± 0.31

**Table 4:** Kinetic parameters of TAD release (K64 polymer matrix).

Sample	k (s^−b^) ± STD	b (-) ± STD	$t_{0}$ (s) ± STD
pure TAD	0.21 ± 0.04	0.99 ± 0.10	0.00 ± 0.19
TAD-K64 PM	0.23 ± 0.03	0.96 ± 0.07	0.11 ± 0.12
TAD-K64 SD (SE)	0.93 ± 0.23	1.71 ± 0.29	0.00 ± 0.13
TAD-K64 SD (SPD)	0.38 ± 0.05	1.20 ± 0.10	0.21 ± 0.09
TAD-K64 SD (HME125)	0.77 ± 0.09	1.06 ± 0.14	0.13 ± 0.08
TAD-K64 SD (HME250)	0.61 ± 0.06	0.85 ± 0.10	0.41 ± 0.09
